# Contrast Sensitivity Comparison of Daily Simultaneous-Vision Center-Near Multifocal Contact Lenses: A Pilot Study

**DOI:** 10.3390/vision9030067

**Published:** 2025-08-01

**Authors:** David P. Piñero, Ainhoa Molina-Martín, Elena Martínez-Plaza, Kevin J. Mena-Guevara, Violeta Gómez-Vicente, Dolores de Fez

**Affiliations:** 1 Group of Optics and Visual Perception, University of Alicante, 03690 Alicante, Spain; david.pinyero@ua.es (D.P.P.); ainhoa.molina@ua.es (A.M.-M.); elena.martinez.plaza@uva.es (E.M.-P.); kjmg92@gmail.com (K.J.M.-G.); vgvicente@ua.es (V.G.-V.); 2 Ophthalmology Unit, Vithas Medimar International Hsopital, 03016 Alicante, Spain; 3 Department of Theoretical Physics, Atomic and Optics, University of Valladolid, 47002 Valladolid, Spain; 4 Alicante Institute for Health and Biomedical Research (ISABIAL), 03010 Alicante, Spain

**Keywords:** chromatic contrast sensitivity, achromatic contrast sensitivity, multifocal contact lens, simultaneous vision, center near, daily contact lens

## Abstract

Our purpose is to evaluate the binocular contrast sensitivity function (CSF) in a presbyopic population and compare the results obtained with four different simultaneous-vision center-near multifocal contact lens (MCL) designs for distance vision under two illumination conditions. Additionally, chromatic CSF (red-green and blue-yellow) was evaluated. A randomized crossover pilot study was conducted. Four daily disposable lens designs, based on simultaneous-vision and center-near correction, were compared. The achromatic contrast sensitivity function (CSF) was measured binocularly using the CSV1000e test under two lighting conditions: room light on and off. Chromatic CSF was measured using the OptoPad-CSF test. Comparison of achromatic results with room lighting showed a statistically significant difference only for 3 cpd (*p* = 0.03) between the baseline visit (with spectacles) and all MCLs. Comparison of achromatic results without room lighting showed no statistically significant differences between the baseline and all MCLs for any spatial frequency (*p* > 0.05 in all cases). Comparison of CSF-T results showed a statistically significant difference only for 4 cpd (*p* = 0.002). Comparison of CSF-D results showed no statistically significant difference for all frequencies (*p* > 0.05 in all cases). The MCL designs analyzed provided satisfactory achromatic contrast sensitivity results for distance vision, similar to those obtained with spectacles, with no remarkable differences between designs. Chromatic contrast sensitivity for the red-green and blue-yellow mechanisms revealed some differences from the baseline that should be further investigated in future studies.

## 1. Introduction

Presbyopia is defined as a physiological condition that causes a progressive decrease in near uncorrected vision due to the loss of accommodative capacity of the ciliary muscle, with clinical manifestations typically occurring after the age of 45. Its prevalence was estimated at 1.8 billion people worldwide in 2015 [1], and this trend is increasing. Aging is the most important factor affecting presbyopia due to the aging population, which leads to an increase in the years lived with presbyopia and, therefore, an increase in the number of presbyopes [2]. In addition to the higher number of presbyopes, aging also causes changes in ocular health, such as a decrease in visual acuity (VA), visual field, contrast sensitivity, and color vision, among others [3].

There are multiple options to correct presbyopia, both surgical and non-surgical. Among the non-surgical options, spectacles remain the most prescribed choice for presbyopes, but in recent years, there has been an increase in contact lens (CL) prescriptions by practitioners [4]. The use of multifocal CLs (MCLs) has grown in clinical practice, and among these, soft simultaneous-vision MCLs are the most popular [5]. Daily disposable lenses are also becoming more common in the general population, and consequently among presbyopic options [6], offering a market niche with high potential. Despite this, practitioners do not see MCLs as a first-choice option, mainly due to the lack of guaranteed success in fitting and the wide range of MCL options on the market, which makes the selection process more challenging. Furthermore, while there are numerous clinical studies evaluating visual performance with MCLs, objective parameters such as visual acuity (VA) do not always reflect patients’ true preferences for one design over another [7].

Contrast sensitivity assessment has been suggested as a complementary evaluation of MCL visual performance, in addition to VA, providing information on how the detection and/or recognition of stimuli is degraded when contrast is reduced due to the different light distribution across retinal focal points caused by the multifocal design. Furthermore, due to the effects of age on the CSF, those presbyopic subjects are more susceptible to degradation not only for achromatic contrast but also for color perception [3] involved in everyday conditions. Surprisingly, despite the large number of studies evaluating the visual performance of MCLs [8,9], only a limited number specifically address achromatic contrast sensitivity [10], and none of them address chromatic vision. Some authors have evaluated the achromatic contrast sensitivity of MCLs in comparison to spectacles [11], single-vision CLs [12], monovision CLs [13,14], EDOF CLs [15], or pinhole CLs [16,17]. Additionally, some authors have compared contrast sensitivity between different MCL designs [18], but such studies remain scarce.

The purpose of the present study was to evaluate the binocular contrast sensitivity function in a presbyopic population and to compare the results obtained with four different simultaneous-vision center-near MCL designs for distance vision under two illumination conditions. Additionally, near CSF was evaluated using a chromatic CSF test to compare visual performance between designs.

## 2. Materials and Methods

### 2.1. Study Design

A randomized crossover pilot study in which each subject acted as their own control was performed, with the order of the MCLs randomized.

### 2.2. Subjects

Participants were recruited from the Optometric Clinic of the University of Alicante (Alicante, Spain). Inclusion criteria required signed informed consent forms in accordance with Good Clinical Practice guidelines and the Declaration of Helsinki. The study protocol was approved by the Institutional Review Board ISABIAL (General Hospital, Alicante, Spain; reference PI2022-052).

Inclusion criteria were the presence of presbyopia (more than +0.50D), willingness to wear CLs and a refractive error compensable with the available CL designs. Exclusion criteria were the presence of pathological findings and any CL intolerance.

### 2.3. Multifocal Contact Lenses

The studied contact lenses (CLs) were all daily disposable, simultaneous-vision, center-near designs: Dailies Total1^®^ Multifocal (Alcon Vision LLC, Fort Worth, TX, USA), MyDay Multifocal (CooperVision Inc., Pleasanton, CA, USA), 1-Day Acuvue Moist Multifocal (Johnson & Johnson Vision Care Inc., Jacksonville, FL, USA), and BioTrue ONEday for Presbyopia (Bausch + Lomb Corp., Rochester, NY, USA). For simplicity, these lenses will hereafter be referred to as Total1, MyDay, Moist, and BioTrue, respectively.

Distance power was determined by the subject’s refractive correction, while the near addition (low, medium, or high) was selected based on the manufacturer’s guidelines and the subjective refraction findings according to normal clinical practice. Each participant wore all four lens designs on separate days, with the order randomized to minimize bias using a random number sequence. The wash-out period was at least one day between visits.

### 2.4. Clinical Protocol

Baseline examination included a full refractive assessment and slit-lamp evaluation to confirm eligibility for contact lens (CL) wear. Study visits involved CL fitting, with outcomes assessed after 30–40 min of adaptation. Variables were analyzed at baseline (with monofocal spectacles) and during four study visits (with multifocal CLs [MCLs]). Primary outcomes included achromatic and chromatic contrast sensitivity function (CSF). Secondary measures comprised distance (3 m, Snellen E chart) and near (40 cm, ETDRS-style Landolt C chart) visual acuity (VA), evaluated at baseline and at each CL fitting visit.

Binocular achromatic CSF was assessed using the CSV-1000 test (VectorVision, Greenville, OH, USA) at 3 m under two lighting conditions: (a) with the room light on (LON); (b) with the room light off (LOFF, with only the light provided by the test). The second condition involved a test luminance level of 85 cd/m^2^ (i.e., manufacturing luminance); therefore, the term LOFF was used operationally to distinguish it from LON. The first condition, hereinafter called LON, involved a test luminance greater than 85 cd/m^2^.

The CSV-1000 evaluates four spatial frequencies (3, 6, 12, and 18 cpd, labeled A–D in the test). For each frequency, eight achromatic contrast steps (test indices 1–8) were presented, with contrast ranges varying by frequency per manufacturer specifications.

Chromatic CSF was measured binocularly using the Optopad-CSF test (40 cm, LOFF). This iPad-based test evaluates contrast thresholds along two chromatic mechanisms: CSF-T (red-green axis) and CSF-D (blue-yellow axis). The test’s validation and design characteristics have been previously reported [19,20]. Both CSF-T and CSF-D assess five spatial frequencies (1, 2, 4, 8, and 12 cpd), each presented in five distinct slides with 16 chromatic contrast steps (test indices 1–16). Contrast ranges varied by spatial frequency, with all stimuli quantified in cone contrast space.

Due to the nature of the CSF measures and the time required to complete all examinations, only a small sample of participants was included for the purpose of this pilot study. All contrast sensitivity measures were performed by the same examiner, who was blinded to the MCL design adapted to the participant.

### 2.5. Statistical Analysis

Statistical analyses were performed using SPSS v.19.0.0 (IBM Corp., Chicago, IL, USA). Due to the small sample size, non-parametric tests were employed. The Friedman test assessed differences between baseline (spectacle correction) and the four multifocal contact lens (MCL) designs. When significant differences were detected (*p* < 0.05), post hoc Wilcoxon signed-rank tests with Bonferroni correction were applied.

Additionally, two summary metrics were calculated to compare CSF performance: area under the curve (AUC) and index of contrast sensitivity (ICS). AUC represents the integrated contrast sensitivity across spatial frequencies, with greater values indicating larger deviations from baseline measurements. ICS quantifies response variability (dispersion relative to mean values), where a higher ICS indicates greater inter-subject variability [21].

## 3. Results

This study included 11 healthy participants (mean age: 51 ± 1 years; range: 47–55 years), comprising 9 women (82%) and 2 men. Table 1 summarizes binocular distance and near visual acuity measurements at baseline and with each multifocal contact lens (MCL) design. The wash-out period was at least one day between visits, and at most one week depending on subjects’ availability.

### 3.1. Achromatic CSF

**Figure 1:**
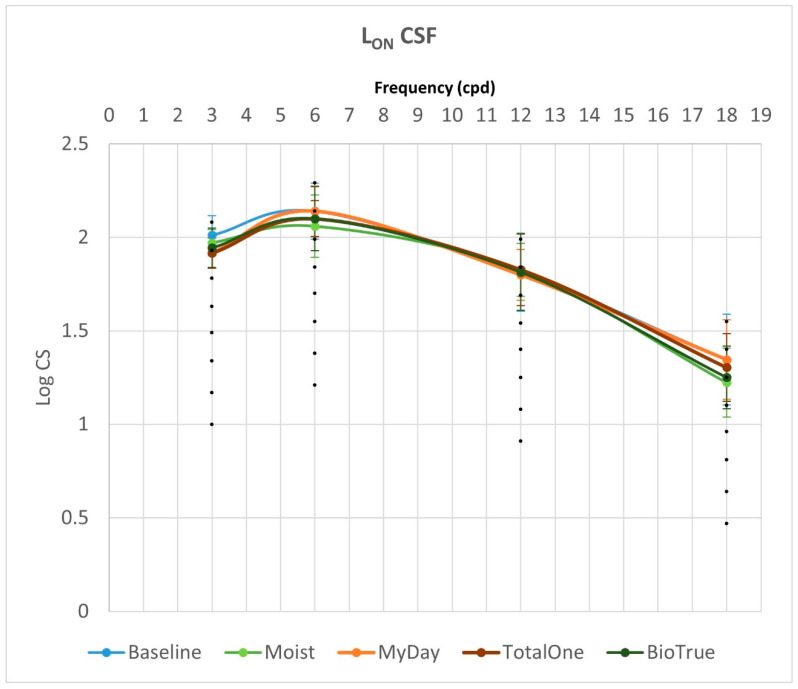
Binocular achromatic contrast sensitivity function (CSF) measurements under L_ON_ condition.

**Figure 2:**
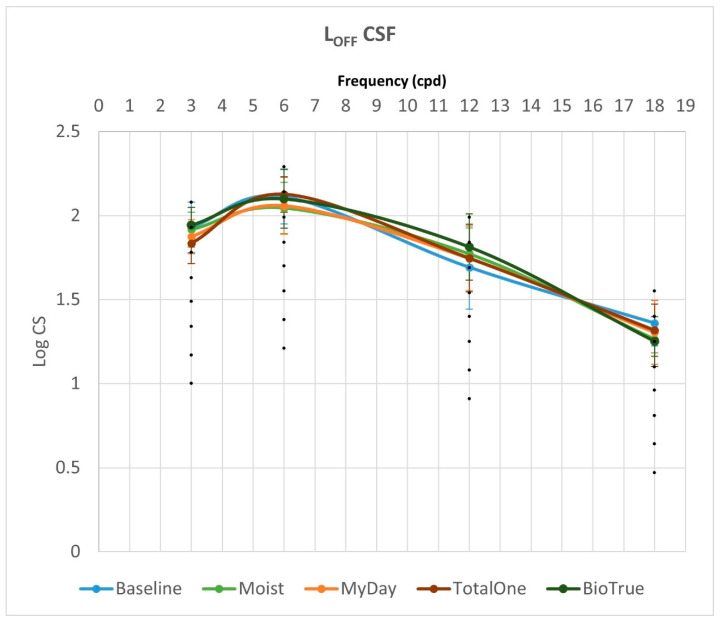
Binocular achromatic contrast sensitivity function (CSF) measurements under L_OFF_ condition.

Figure 1 and Figure 2 summarize binocular achromatic contrast sensitivity function (CSF) measurements under L_ON_ and L_OFF_ photopic conditions, respectively, comparing baseline values with those obtained using each multifocal contact lens (MCL) design.

L_ON_ achromatic CSF analysis revealed no significant differences between baseline and any MCL design (Moist, MyDay, Total1, BioTrue) at 6, 12, or 18 cpd (all *p* > 0.05). However, a significant difference was observed at 3 cpd (*p* = 0.03). Post hoc analysis at 3 cpd showed that MyDay performed significantly worse than baseline (*p* = 0.02), though this difference became non-significant after Bonferroni correction (*p* = 0.08). When comparing only MCL designs (excluding baseline), no significant differences were found at any spatial frequency (all *p* > 0.05).

Under L_OFF_ conditions, no significant differences were observed between baseline and any MCL design (Moist, MyDay, Total1, BioTrue) at any spatial frequency (all *p* > 0.05). When excluding baseline measurements, significant differences emerged between MCL designs at 3 cpd (*p* = 0.026). Post hoc analysis revealed that BioTrue demonstrated superior performance to Total1 at 3 cpd (*p* = 0.023), though this difference did not survive Bonferroni correction (adjusted *p* = 0.136). Complete achromatic CSF results for both L_ON_ and L_OFF_ photopic conditions, including all pairwise comparisons (with and without baseline), are presented in Table 2.

While L_OFF_ CSF values were consistently lower than L_ON_ values for both baseline and all MCL designs, these reductions were not statistically significant at any spatial frequency (all *p* > 0.05). Similarly, AUC comparisons between L_ON_ and L_OFF_ conditions revealed no significant differences among MCL designs (all *p* ≥ 0.152). The ICS also showed no significant variations, either under L_ON_ or L_OFF_ conditions (all *p* ≥ 0.055).

### 3.2. Chromatic CSF

Binocular chromatic contrast sensitivity function (CSF) measurements, comparing baseline values with multifocal contact lens (MCL) performance, are presented separately for the two chromatic mechanisms: CSF-T (red-green) in Table 3 and CSF-D (blue-yellow) in Table 4. The CSF-T results showed comparable values to achromatic CSF measurements, while CSF-D values were clinically significantly lower.

Analysis of CSF-T revealed no significant differences from the baseline and between MCL designs (Moist, MyDay, Total1, BioTrue) at 1, 2, 8, or 12 cpd (all *p* > 0.05). However, at 4 cpd, we observed significant overall variation (*p* = 0.002). Post hoc analysis at 4 cpd showed that MyDay performed similarly to baseline (*p* = 0.07). Moist, Total1, and BioTrue demonstrated significantly reduced sensitivity versus baseline (*p* = 0.011, *p* = 0.017, and *p* = 0.003, respectively). After Bonferroni correction, only Moist (adjusted *p* = 0.044) and BioTrue (adjusted *p* = 0.012) maintained significance. When comparing only MCL designs (excluding baseline), no significant differences emerged at any spatial frequency (all *p* > 0.05).

Analysis of CSF-D (blue-yellow mechanism) revealed no statistically significant differences between baseline and any MCL design (Moist, MyDay, Total1, BioTrue) at any tested spatial frequency (1, 2, 4, 8, or 12 cpd; all *p* > 0.05). Similarly, when comparing only the MCL designs (excluding baseline), no significant differences were observed at any spatial frequency (all *p* > 0.05).

Comparative analysis revealed no significant differences in AUC measurements among MCL designs for either CSF-T or CSF-D (all *p* ≥ 0.301). Similarly, the ICS showed no significant variations across lens designs in both chromatic tests (all *p* ≥ 0.896).

## 4. Discussion

Comparing multifocal contact lens (MCL) performance presents inherent challenges due to substantial design variability. These difficulties are compounded when evaluating contrast sensitivity, given the diversity of available assessment methods. Various contrast sensitivity function (CSF) tests—including FACT, VCTS, and CSV-1000 [10]—employ different stimulus contrasts and spatial frequency ranges, making direct cross-study comparisons problematic. Notably, the CSV-1000 test used in this pilot study was specifically developed for distance vision assessment under real-world viewing conditions and has been validated for clinical applications [21,22].

In this pilot study, achromatic contrast sensitivity was evaluated using the CSV-1000 test. The results demonstrated that multifocal contact lenses (MCLs) maintained comparable CSF performance to monofocal spectacle correction, with no statistically or clinically significant reductions observed across spatial frequencies. All measurements—both baseline and MCL conditions—consistently fell within the upper response range of the test, indicating excellent achromatic contrast sensitivity preservation with all lens designs. While minor reductions in median values were noted for low spatial frequencies (particularly with MyDay), these differences were neither statistically significant (*p* > 0.05) nor clinically meaningful. The observed preservation of achromatic contrast sensitivity may be partially explained by a ceiling effect, as measurements approached the upper detection limit of the CSV-1000 test, reducing the range of possible responses [21,22]. This potential measurement limitation aligns with previous reports questioning the test’s sensitivity for discriminating high-performance visual function [21].

Previous studies report conflicting findings regarding MCLs’ impact on achromatic contrast sensitivity. Llorente-Guillemot et al. (2012) [11] found significantly reduced CSF at all spatial frequencies with PureVision multifocal (high-add) lenses compared to progressive addition lenses (PALs) using the FACT-FVA test under simulated distance conditions. Conversely, Fernandes et al. (2013) [13] reported no significant CSF reduction with Biofinity multifocal lenses versus various baseline corrections (PALs, monofocal spectacles, or single-vision CLs) using the same test protocol. While these studies provide valuable context, direct comparison with present findings is limited by three key methodological differences: (1) distinct MCL designs evaluated, (2) varied baseline comparators, and (3) different CSF assessment methods (FACT-FVA vs. CSV-1000). Notably, both prior studies employed simulated distance testing conditions, whereas in this study, achromatic CSF was measured under real-world viewing conditions.

While all studied MCLs employed simultaneous-vision center-near designs, subtle variations in optical zone geometry and power profile transitions led to minor, non-significant differences in spatial frequency performance. These variations were neither statistically significant (all *p* > 0.05) nor clinically meaningful. Direct literature comparisons remain challenging due to (1) limited published CSF data on these specific lens designs, and (2) methodological differences in reporting. However, previous CSV-1000 studies of other MCL designs show similar trends. Piñero et al. (2015) [18] found no significant CSF differences among three multifocal designs (Biofinity, Air Optix, and Duette) and Martinez-Alberquilla et al. (2021) [15] reported comparable CSF between Biofinity multifocal and EDOF lenses. Notably, these studies presented results graphically rather than numerically, precluding direct quantitative comparison with present findings. This underscores the need for standardized reporting metrics in CSF research.

A critical gap in MCL evaluation remains the limited assessment of near vision contrast sensitivity. While specialized near CSF tests exist, they are rarely employed in either clinical practice or research settings. The few studies examining near CSF with MCLs [11,14,16,17] have not statistically compared near versus distance performance. In this study, direct distance–near comparisons were precluded by fundamental methodological differences: achromatic assessment of distance CSF (CSV-1000) and chromatic evaluation of near CSF (Optopad-CSF). This discrepancy highlights the need for standardized, comparable CSF protocols that can evaluate both distance and near vision under consistent testing conditions (e.g., same chromatic/achromatic parameters, equivalent luminance levels). Such standardization would enable meaningful comparisons of MCL performance across different viewing distances.

Another limitation found in clinical practice by clinicians is the variability in the near addition steps between designs. The same addition on trial lenses agrees with different step recommendations depending on the manufacturer, and this causes near addition induced by multifocal design to differ between MCLs. These differences could affect the VA and CSF results when comparing different designs on the same subject due to the higher or lower power of the near zone, but in order to compare MCL designs in clinical practice, each addition and power was selected according to recommendations provided by manufacturers, as occurs in real clinical environments. The purpose of this study was to compare the visual function results of each subject, adapted with the MCLs recommended in clinical practice, and to analyze whether the recommended MCL designs provide better or worse results than the others in terms of VA and CSF.

### 4.1. L_ON_ vs. L_OFF_ Achromatic Measurements

The lighting conditions during contrast sensitivity testing significantly impact visual performance measurements. The CSV-1000 test’s inherent luminance establishes photopic testing conditions regardless of ambient room lighting. While some studies operationally define ‘mesopic’ conditions as testing with room lights off (versus on), this does not meet strict mesopic criteria. The mesopic vision is set below 1 cd/m^2^, but in practice, this value is exceeded only by keeping the CSV-1000 test on, which emits 85 cd/m^2^. For clinical clarity in this study, the following definitions were used: L_ON_ conditions with test illumination and room lighting, and L_OFF_ conditions with test illumination only. This distinction provides a practical framework for comparing relative performance under different lighting scenarios in clinical settings since real mesopic conditions are unfeasible with an illuminated test such as CSV-1000.

Previous studies have evaluated achromatic contrast sensitivity with multifocal contact lenses (MCLs) under different lighting conditions [11,15,16,17], though none have statistically compared performance between these lighting conditions. Martinez-Alberquilla et al. (2021) [15] and García-Lázaro et al. (2014) [17] show generalized contrast sensitivity reductions in mesopic conditions. García-Lázaro et al. (2013) [16] report more pronounced mesopic reductions at higher spatial frequencies (though without statistical verification). Llorente-Guillemot et al. (2012) [11] observed similar mesopic reductions for both MCLs and spectacles at high spatial frequencies.

The present pilot study extends these findings by formally analyzing differences between light conditions, revealing no statistically significant variations despite numerical decreases in measurements. This aligns with Llorente-Guillemot’s non-significant MCL–spectacle comparisons under mesopic conditions. The limited lighting-dependent effects may stem from two factors: testing conditions may not achieve true mesopic retinal adaptation, as retinal mechanisms remain photopically dominated without complete dark adaptation, and the clinical testing paradigm (brief exposures without full dark adaptation) may not sufficiently engage mesopic visual pathways. These findings suggest that for typical clinical lighting conditions, MCL performance remains stable across illumination changes, with any observed differences being subclinical in nature.

### 4.2. Chromatic CSF

While previous studies have evaluated near achromatic contrast sensitivity with MCLs, to the best of our knowledge, this represents the first comprehensive assessment of chromatic contrast sensitivity, comparing both multiple MCL designs and monofocal spectacle correction. Using the validated Optopad-CSF test [19,20], we specifically evaluated two chromatic mechanisms: red-green (CSF-T) and blue-yellow (CSF-D). Baseline measurements aligned with established normative values for each age group [20,23], showing the expected pattern of higher sensitivity in the red-green (CSF-T) channel and reduced sensitivity in the more vulnerable blue-yellow (CSF-D) channel. This age-appropriate normative comparison is particularly relevant given the known decline in chromatic vision with aging [23], and confirms that the present study population exhibited typical chromatic contrast sensitivity for their age group prior to MCL evaluation.

For the red-green chromatic mechanism (CSF-T), distinct spatial frequency-dependent performance patterns were observed. Regarding the MCLs vs. baseline comparison, there was a significant reduction at 4 cpd for Moist (*p* = 0.011) and BioTrue (*p* = 0.003) vs. spectacles, a comparable performance at low–medium spatial frequencies (1–2 cpd), and consistent (though non-significant) baseline superiority at high frequencies (8–12 cpd). In the case of inter-CL comparisons, MyDay showed numerically superior performance at high frequencies, BioTrue demonstrated the lowest high-frequency sensitivity, and there were no significant differences between designs (all *p* > 0.05 after correction). The 4 cpd significance (uncorrected *p* < 0.05) suggests potential mid-frequency chromatic sensitivity trade-offs with certain MCL designs, though the clinical relevance appears limited given (a) non-significance after multiple comparison correction, and (b) preserved performance at other frequencies.

The blue-yellow chromatic mechanism (CSF-D) consistently demonstrated reduced sensitivity compared to both achromatic and red-green (CSF-T) mechanisms across all conditions (baseline and MCL designs), confirming previous reports [20,23]. However, several methodological considerations warrant caution in interpreting these results. One of them is the measurement variability, with exceptionally high inter-subject variability (SD > mean in some cases), and characteristically low absolute sensitivity values for the S-cone pathway. There are also statistical limitations that include the reduced statistical power from the small sample size (*n* = 11), the floor effects potentially obscuring true differences, and the increased susceptibility to outlier influence. From the clinical perspective, relative performance patterns were maintained despite variability, rank-order consistency was preserved across lens designs, and the observed trends were aligned with known blue-yellow pathway vulnerability. These findings suggest that while the blue-yellow mechanism shows predictable sensitivity reductions, the CSV-1000 test paradigm may lack sufficient resolution to detect subtle MCL-induced variations in this already-compromised chromatic channel, particularly with limited sample sizes.

Despite the lack of statistically significant results, it should be noted that blue-yellow sensitivity CSF-D with MCLs was worse than that obtained at baseline for low and medium spatial frequencies, but this tendency was reversed for high spatial frequencies where MCLs showed better results than baseline. These results could indicate that for low spatial frequencies, the studied MCLs are associated with some kind of disadvantage compared to spectacles, and on the other hand, offer some kind of advantage for high spatial frequencies compared to spectacles. It should be noted that all the MCL designs studied in the present study were blue-tinted CLs, and this color could be acting as a blue filter and therefore could be affecting the response of blue-yellow sensitivity as occurs with other color filters [24]. This tendency should be explored in future studies with other non-tinted CLs to confirm. Additionally, higher sample sizes could confirm the tendencies found in this case, and in the whole analysis.

## 5. Conclusions

All MCL designs analyzed in the present pilot study provided satisfactory achromatic contrast sensitivity results for distance vision, similar to spectacles, and with no remarkable differences between designs. Despite a small non-significant reduction in achromatic contrast sensitivity in room-light-off conditions, all studied MCL designs provide contrast sensitivity results comparable to spectacles with no differences between designs. Chromatic contrast sensitivity for red-green and blue-yellow mechanisms revealed some differences with the baseline that should be better investigated in future studies.

## Figures and Tables

**Table 1:** Mean (SD), median, and range values for distance and near vision obtained at baseline and with MCLs. Note that baseline VA was measured with ophthalmic lenses correcting distance refraction for distance vision, and near refraction for near vision.

	Distance VA	Near VA
Baseline	−0.11 (0.01) −0.10 (−0.18 to −0.10)	−0.02 (0.01) 0.00 (−0.10 to 0.00)
Moist	−0.04 (0.03) −0.04 (−0.18 to 0.12)	0.12 (0.05) 0.10 (−0.10 a 0.48)
MyDay	−0.05 (0.02) −0.06 (−0.16 to 0.02)	0.14 (0.06) 0.14 (−0.20 a 0.49)
Total1	−0.06 (0.02) −0.08 (−0.18 to 0.06)	0.17 (0.06) 0.20 (−0.18 a 0.46)
Biotrue	−0.10 (0.02) −0.08 (−0.18 to 0.02)	0.31 (0.08) 0.14 (0.00 a 0.80)

**Table 2:** Mean (SD), median, and range values for achromatic CSF on a logarithmic scale obtained binocularly for L_ON_ and L_OFF_ conditions with the MCL designs. The *p*-values from the Friedman analysis including (up) and excluding (down) the baseline visit are provided.

	Frequency (cpd)	Baseline	Moist	MyDay	Total1	BioTrue	p_baseline_ p_MCL_
L_ON_ CSF	3	2.01 (0.10) 2.08 (1.78 to 2.08)	1.97 (0.07) 1.93 (1.93 to 2.08)	1.92 (0.08) 1.93 (1.78 to 2.08)	1.92 (0.08) 1.93 (1.78 to 2.08)	1.94 (0.11) 1.93 (1.78 to 2.08)	0.030 0.194
	6	2.14 (0.15) 2.14 (1.84 to 2.29)	2.06 (0.17) 2.14 (1.70 to 2.29)	2.14 (0.13) 2.14 (1.84 to 2.29)	2.10 (0.10) 2.14 (1.99 to 2.29)	2.12 (0.17) 2.14 (1.84 to 2.29)	0.214 0.217
	12	1.81 (0.21) 1.84 (1.25 to 1.99)	1.83 (0.14) 1.84 (1.54 to 1.99)	1.79 (0.14) 1.84 (1.54 to 1.99)	1.83 (0.19) 1.84 (1.40 to 1.99)	1.76 (0.20) 1.84 (1.40 to 1.99)	0.879 0.707
	18	1.35 (0.24) 1.40 (0.81 to 1.55)	1.22 (0.19) 1.25 (0.81 to 1.55)	1.35 (0.21) 1.40 (0.96 to 1.55)	1.30 (0.18) 1.25 (1.10 to 1.55)	1.35 (0.17) 1.25 (1.10 to 1.55)	0.277 0.135
L_OFF_ CSF	3	1.93 (0.15) 1.93 (1.63 to 2.08)	1.92 (0.11) 1.93 (1.78 to 2.08)	1.88 (0.10) 1.93 (1.63 to 1.93)	1.83 (0.12) 1.93 (1.63 to 1.93)	1.94 (0.11) 1.93 (1.78 to 2.08)	0.094 **0.026**
	6	2.11 (0.16) 2.14 (1.84 to 2.29)	2.05 (0.15) 1.99 (1.70 to 2.29)	2.06 (0.17) 2.14 (1.84 to 2.29)	2.13 (0.11) 2.14 (1.99 to 2.29)	2.10 (0.18) 2.14 (1.70 to 2.29)	0.330 0.346
	12	1.69 (0.25) 1.69 (1.25 to 1.99)	1.77 (0.16) 1.84 (1.54 to 1.99)	1.75 (0.19) 1.84 (1.40 to 1.99)	1.75 (0.20) 1.84 (1.40 to 1.99)	1.81 (0.20) 1.84 (1.40 to 1.99)	0.403 0.284
	18	1.36 (0.14) 1.25 (1.25 to 1.55)	1.26 (0.08) 1.25 (1.10 to 1.40)	1.31 (0.19) 1.40 (0.96 to 1.55)	1.32 (0.16) 1.25 (1.10 to 1.55)	1.25 (0.15) 1.25 (0.96 to 1.55)	0.416 0.755

**Table 3:** Mean (SD), median, and range values for chromatic red-green CSF on a logarithmic scale obtained binocularly for the CSF-T test at baseline and with the MCL designs. The *p*-values from the Friedman analysis including (up) and excluding (down) the baseline visit are provided.

	Frequency (cpd)	Baseline	Moist	MyDay	Total1	BioTrue	p_baseline_ p_MCL_
CSF-T	1	2.32 (0.21) 2.48 (2.06 to 2.48)	2.40 (0.17) 2.48 (2.06 to 2.48)	2.36 (0.20) 2.48 (2.06 to 2.48)	2.36 (0.20) 2.48 (2.06 to 2.48)	2.40 (0.17) 2.48 (2.06 to 2.48)	0.856 0.909
	2	2.42 (0.18) 2.48 (1.88 to 2.48)	2.44 (0.13) 2.48 (2.06 to 2.48)	2.33 (0.25) 2.48 (1.88 to 2.48)	2.32 (0.21) 2.48 (2.06 to 2.48)	2.40 (0.17) 2.48 (2.05 to 2.48)	0.554 0.481
	4	2.14 (0.23) 2.06 (1.88 to 2.48)	1.86 (0.16) 1.88 (1.59 to 2.06)	1.90 (0.24) 1.88 (1.59 to 2.48)	1.81 (0.22) 1.88 (1.46 to 2.06)	1.77 (0.12) 1.76 (1.59 to 1.88)	0.002 0.253
	8	1.87 (0.26) 1.88 (1.46 to 2.48)	1.79 (0.10) 1.76 (1.66 to 1.88)	1.84 (0.18) 1.88 (1.59 to 2.06)	1.77 (0.17) 1.76 (1.46 to 2.05)	1.63 (0.48) 1.88 (0.31 to 2.05)	0.516 0.449
	12	1.79 (0.23) 1.88 (1.37 to 2.06)	1.60 (0.15) 1.59 (1.41 to 1.88)	1.67 (0.19) 1.66 (1.37 to 1.88)	1.63 (0.22) 1.59 (1.29 to 2.06)	1.56 (0.14) 1.52 (1.37 to 1.88)	0.056 0.205

**Table 4:** Mean (SD), median, and range values for chromatic blue-yellow CSF on a logarithmic scale obtained binocularly for the CSF-D test at baseline and with the MCL designs. The *p*-values including (up) and excluding (down) the baseline visit are provided.

	Frequency (cpd)	Baseline	Moist	MyDay	Total1	BioTrue	p_baseline_ p_MCL_
CSF-D	1	0.91 (0.17) 0.96 (0.78 to 1.38)	0.74 (0.21) 0.66 (0.49 to 0.96)	0.74 (0.20) 0.78 (0.42 to 0.96)	0.69 (0.14) 0.66 (0.42 to 0.96)	0.75 (0.20) 0.66 (0.56 to 0.96)	0.149 0.841
	2	0.81 (0.31) 0.78 (0.49 to 1.38)	0.66 (0.31) 0.66 (0.36 to 1.38)	0.66 (0.17) 0.66 (0.31 to 0.96)	0.53 (0.20) 0.49 (0.19 to 0.78)	0.65 (0.24) 0.66 (0.36 to 0.96)	0.110 0.274
	4	0.34 (0.19) 0.31 (0.09 to 0.66)	0.27 (0.11) 0.27 (0.12 to 0.49)	0.33 (0.15) 0.31 (0.09 to 0.56)	0.28 (0.18) 0.27 (0.05 to 0.66)	0.24 (0.10) 0.22 (0.12 to 0.49)	0.508 0.515
	8	0.10 (0.12) 0.05 (0.05 to 0.42)	0.18 (0.11) 0.18 (0.05 to 0.42)	0.18 (0.13) 0.19 (0.05 to 0.42)	0.20 (0.15) 0.22 (0.05 to 0.48)	0.20 (0.14) 0.19 (0.05 to 0.36)	0.121 0.796
	12	0.08 (0.11) 0.05 (0.05 to 0.42)	0.09 (0.06) 0.05 (0.05 to 0.22)	0.11 (0.11) 0.05 (0.05 to 0.42)	0.13 (0.12) 0.05 (0.05 to 0.42)	0.08 (0.05) 0.05 (0.05 to 0.19)	0.378 0.828

## Data Availability

Raw data were generated at the University of Alicante. Derived data supporting the findings of this study are available from the corresponding author, D.d.F., on reasonable request.

## Abbreviations

The following abbreviations are used in this manuscript:

CL	contact lens
MCL	multifocal contact lens
CSF	contrast sensitivity function
CSF-T	contrast sensitivity function of chromatic mechanisms for red-green axis
CSF-D	contrast sensitivity function of chromatic mechanisms for blue-yellow axis
AUC	area under the curve
ICS	index of contrast sensitivity
L_ON_	room light on
L_OFF_	room light off

## References

[1] Fricke T.R., Tahhan N., Resnikoff S., Papas E., Burnett A., Ho S.M., Naduvilath T., Naidoo K.S. (2018). Global Prevalence of Presbyopia and Vision Impairment from Uncorrected Presbyopia: Systematic Review, Meta-analysis, and Modelling. Ophthalmology.

[2] Li S., Ye E., Huang J., Wang J., Zhao Y., Niu D., Yue S., Huang X., Liu J., Hou X. (2022). Global, regional, and national years lived with disability due to blindness and vision loss from 1990 to 2019: Findings from the Global Burden of Disease Study 2019. Front. Public Health.

[3] Erdinest N., London N., Lavy I., Morad Y., Levinger N. (2021). Vision through Healthy Aging Eyes. Vision.

[4] Rueff E.M., Bailey M.D. (2017). Presbyopic and non-presbyopic contact lens opinions and vision correction preferences. Contact Lens Anterior Eye.

[5] Morgan P.B., Woods C.A., Tranoudis I.G., Efron N., Jones L., Faccia P., Rivadeneira D., Grupcheva C.N., Jones D., Cely L.M.R. (2025). International Contact Lens Prescribing in 2024. Contact Lens Spectr..

[6] Efron N., Morgan P.B., Woods C.A., International Contact Lens Prescribing Survey Consortium (2013). An international survey of daily disposable contact lens prescribing. Clin. Exp. Optom..

[7] Jong M., Tilia D., Sha J., Diec J., Thomas V., Bakaraju R.C. (2019). The Relationship between Visual Acuity, Subjective Vision, and Willingness to Purchase Simultaneous-image Contact Lenses. Optom. Vis. Sci..

[8] Pérez-Prados R., Piñero D.P., Pérez-Cambrodí R.J., Madrid-Costa D. (2017). Soft multifocal simultaneous image contact lenses: A review. Clin. Exp. Optom..

[9] Molina-Martín A., Piñero D.P., Martínez-Plaza E., Rodríguez-Vallejo M., Fernández J.M. (2023). Efficacy of presbyopia-correcting contact lenses: A systematic review. Eye Contact Lens.

[10] Mena-Guevara K.J.M., de Fez D., Piñero D.P. (2024). Impact on Distance and Near Contrast Sensitivity of Multifocal Contact Lenses: A Systematic Review. Eye Contact Lens.

[11] Llorente-Guillemot A., García-Lazaro S., Ferrer-Blasco T., Perez-Cambrodi R.J., Cerviño A. (2012). Visual performance with simultaneous vision multifocal contact lenses. Clin. Exp. Optom..

[12] García-Lázaro S., Ferrer-Blasco T., Madrid-Costa D., Albarrán-Diego C., Montés-Micó R. (2015). Visual performance of four simultaneous-image multifocal contact lenses under dim and glare conditions. Eye Contact Lens.

[13] Fernandes P.R., Neves H.I.F., Lopes-Ferreira D.P., Jorge J.M., González-Meijome J.M. (2013). Adaptation to multifocal and monovision contact lens correction. Optom. Vis. Sci..

[14] Gupta N., Naroo S.A., Wolffsohn J.S. (2009). Visual comparison of multifocal contact lens to monovision. Optom. Vis. Sci..

[15] Martínez-Alberquilla I., García-Montero M., Ruiz-Alcocer J., Crooke A., Madrid-Costa D. (2021). Visual function, ocular surface integrity and symptomatology of a new extended depth-of-focus and a conventional multifocal contact lens. Contact Lens Anterior Eye.

[16] García-Lázaro S., Albarrán-Diego C., Ferrer-Blasco T., Radhakrishnan H., Montés-Micó R. (2013). Visual performance comparison between contact lens-based pinhole and simultaneous vision contact lenses. Clin. Exp. Optom..

[17] García-Lázaro S., Ferrer-Blasco T., Radhakrishnan H., Albarrán-Diego C., Montés-Micó R. (2014). Artificial pupil versus contralateral balanced contact lens fit for presbyopia correction. Arq. Bras. Oftalmol..

[18] Piñero D.P., Carracedo G., Ruiz-Fortes P., Pérez-Cambrodí R.J. (2015). Comparative analysis of the visual performance and aberrometric outcomes with a new hybrid and two silicone hydrogel multifocal contact lenses: A pilot study. Clin. Exp. Optom..

[19] de Fez D., Luque M.J., Matea L., Piñero D.P., Camps V.J. (2018). New iPAD-based test for the detection of color vision deficiencies. Graefe’s Arch. Clin. Exp. Ophthalmol..

[20] de Fez D., García C., Luque-Cobija M.J., Mena-Guevara K.J., Daudén P., Piñero D.P. (2024). Validation of a New Test for Measuring the Contrast Sensitivity Function (Optopad-CSF) at Near Vision. Diagnostics.

[21] Koefoed V.F., Baste V., Roumes C., Høvding G. (2015). Contrast sensitivity measured by two different test methods in healthy, young adults with normal visual acuity. Acta Ophthalmol..

[22] Kelly S.A., Pang Y., Klemencic S. (2012). Reliability of the CSV-1000 in adults and children. Optom. Vis. Sci..

[23] Mena-Guevara K.J., Piñero D.P., Luque M.J., de Fez D. (2024). The Measurement of Contrast Sensitivity in Near Vision: The Use of a Digital System vs. a Conventional Printed Test. Technologies.

[24] de Fez M.D., Luque M.J., Viqueira V. (2002). Enhancement of contrast sensitivity and losses of chromatic discrimination with tinted lenses. Optom. Vis. Sci..

